# Hyperandrogenism and Cardiometabolic Risk in Pre- and Postmenopausal Women—What Is the Evidence?

**DOI:** 10.1210/clinem/dgad590

**Published:** 2023-10-27

**Authors:** Angelica Lindén Hirschberg

**Affiliations:** Department of Women's and Children's Health, Karolinska Institutet and Department of Gynecology and Reproductive Medicine, Karolinska University Hospital, SE-171 76 Stockholm, Sweden

**Keywords:** hyperandrogenism, polycystic ovary syndrome, cardiovascular risk factors, type 2 diabetes, hypertension, dyslipidemia, metabolic syndrome, cardiovascular disease, coronary artery disease, stroke, cardiovascular mortality, premenopausal women, postmenopausal women

## Abstract

Hyperandrogenism in women, such as polycystic ovary syndrome, ovarian hyperthecosis, congenital adrenal hyperplasia, and androgen-secreting tumors, are all associated with increased prevalence of cardiovascular risk factors that include type 2 diabetes, hypertension, dyslipidemia, and metabolic syndrome. However, it is not clear whether this also implies enhanced risk of cardiovascular disease and mortality. Furthermore, the involvement of obesity and menopausal status for cardiometabolic risk in these women has not been elucidated. Based on the most recent systematic reviews and meta-analyses, this review summarizes the latest scientific evidence. To conclude, hyperandrogenism in premenopausal women is associated with enhanced prevalence of cardiovascular risk factors, as well as increased risk of cardiovascular disease and mortality, independently of body mass index. In contrast, elevated cardiovascular risk factors and increased risk of myocardial infarction and stroke in hyperandrogenic postmenopausal women are dependent on obesity. Furthermore, the overall risk of cardiovascular disease and coronary artery disease in hyperandrogenic postmenopausal women is similar to controls. The reason for a reduced cardiometabolic risk after menopause in hyperandrogenic women compared to nonhyperandrogenic women is not clear. It can be speculated that the difference in endocrine balance and metabolic status between women with and without hyperandrogenism might decrease after menopause because hyperandrogenism usually improves with age, whereas menopausal transition itself is associated with androgen dominance and abdominal obesity. Although we have gained increased knowledge about cardiometabolic risks in women with hyperandrogenism, it must be acknowledged that the quality of data is overall low. More research is needed, especially longer and larger follow-up studies in women with hyperandrogenism of different etiologies and phenotypes.

Hyperandrogenism, defined as androgen excess originating from the ovaries and/or the adrenal glands, is a common condition in premenopausal women, and may also persist or develop in postmenopausal women. The symptoms include hirsutism, acne, and androgenic alopecia, and in severe and more rare cases, virilizing symptoms, also including deepening of the voice, breast atrophy, increased muscle mass, and enlargement of the clitoris (1).

The present review focuses on the clinical diagnoses of hyperandrogenism described later in the article. However, references are also made to associations between testosterone levels, or derived measures such as free androgen index (FAI; 100 × total testosterone SHBG) and cardiometabolic outcomes. In this context, it should be acknowledged that the methodology of testosterone determination has varied among studies and today only liquid chromatography-tandem mass spectrometry is considered accurate, whereas immunoassays are burdened with cross-reactivity and are not sensitive enough for determination at low concentrations (2). Furthermore, although FAI is recommended for clinical use in the diagnosis of polycystic ovary syndrome (PCOS) (3), it is a derived measure of testosterone that lacks certified reference ranges and may reflect changes in SHBG as well as testosterone.

PCOS is considered the most common cause of hyperandrogenism in women and is characterized by anovulation, hyperandrogenism, and polycystic ovarian morphology (4, 5). Based on the Rotterdam definition of PCOS, the global prevalence in premenopausal women ranges between 10% and 13% (3, 6). A history of oligo/amenorrhea and ovarian hyperandrogenism during the reproductive years can be used for diagnosing PCOS in postmenopausal women, as suggested by The Endocrine Society Clinical Practice Guideline (7). Although symptoms of hyperandrogenism in PCOS are relatively mild to moderate and often improve after menopause, it is recognized that postmenopausal women with a history of PCOS are more likely to have elevated serum androgen levels (total testosterone, FAI, androstenedione) and higher prevalence of hirsutism than women without PCOS (8, 9). Ovarian hyperthecosis is considered the peri- and postmenopausal woman's variant of PCOS. It is caused by nests of luteinized theca cells in the ovarian stroma, producing excess amounts of testosterone (1). Ovarian hyperthecosis is a rare disorder and the symptoms of hyperandrogenism are usually more extreme with slow progression to virilization, which is not the case in PCOS (1, 10).

Other hyperandrogenic conditions include congenital adrenal hyperplasia (CAH) and androgen-producing tumors. CAH is an autosomal recessive disease caused by an enzyme deficiency in the adrenal cortex steroid biosynthesis, leading to low or absent production of cortisol with concomitant overproduction of adrenal androgens (11). The nonclassic variant of CAH has a prevalence of 1 of 1000 to 2000 individuals, is associated with mild symptoms of hyperandrogenism very similar to PCOS, and should be considered also in postmenopausal women with hyperandrogenism (1, 12). Classic CAH, on the other hand, is rare (1 of 10 000 to 20 000) and usually diagnosed in infancy because of varying degrees of virilization in females, or in the most severe cases because of life-threatening salt wasting (13). Furthermore, androgen-secreting tumors of either ovarian or adrenal origin are very rare and will cause symptoms of hyperandrogenism in both pre- and postmenopausal women (1, 14, 15). The clinical presentation differs from other hyperandrogenic conditions by the rapid progress of severe hyperandrogenism to virilization. In particular, the risk of malignancy of adrenal tumors should be considered (16).

Hyperandrogenic disorders, independently of cause, is strongly associated with metabolic disorders, including insulin resistance, accumulation of abdominal fat, and obesity (17-20). Furthermore, in women with hyperandrogenism, there is an increased risk of type 2 diabetes and metabolic syndrome (ie, the cluster of abdominal obesity, dyslipidemia, hyperglycemia, and hypertension) (17, 18). In turn, type 2 diabetes and metabolic syndrome are potent risk factors for atherosclerotic cardiovascular disease (21). However, there is considerable controversy as to whether hyperandrogenism also entails an enhanced risk of cardiovascular events and mortality (22, 23).

Cardiovascular disease that includes ischemic heart disease is the leading cause of death in women globally, accounting for 35% of total deaths in women (24). The global age-standardized cardiovascular disease mortality in women 2019 was estimated at 204 deaths per 100 000 (24). It is well known that the incidence of cardiovascular disease increases after menopause independently of age (25), which indicates that the pathogenesis is dependent on sex hormone status (26). Endogenous estrogen is known to be cardioprotective by promoting endothelial function, vasodilation, healthy blood pressure, favorable blood lipid profile, and insulin sensitivity (27, 28). In contrast, studies indicate that increased androgenicity, as shown by FAI, is associated with cardiovascular risk factors in postmenopausal women (29-34), whereas corresponding associations with total testosterone have yielded conflicting results (29, 31, 34-38). During normal menopausal transition, estradiol levels gradually decline, whereas ovarian androgen production declines by age and does not change specifically in relation to menopause (39, 40). The latter could be explained by a decrease in ovarian theca cell androgen production, whereas stroma cell production of androgens is increased because of enhanced stimulation by LH (1). The physiological shift from estrogen dominance to a dominance of androgens during menopausal transition might be of importance for enhanced cardiovascular risk after menopause.

This review aims to clarify the evidence for possible associations between hyperandrogenic disorders and cardiometabolic risk factors, cardiovascular disease, and mortality, respectively, and to investigate the role of obesity and menopause for such associations. Literature research of primarily systematic reviews and meta-analyses was performed up to July 2023 in PubMed, Google Scholar, and Cochrane databases. The terms used were “hyperandrogenism” OR “PCOS” OR “CAH” OR “ovarian hyperthecosis” OR “androgen-secreting tumor” AND “insulin resistance” OR “obesity” OR “type 2 diabetes” OR “cardiovascular risk factors” OR “hypertension” OR “hyperlipidemia” OR “metabolic syndrome“ OR “cardiovascular disease” OR “myocardial infarction” OR “coronary heart disease” OR “stroke” OR “cardiovascular mortality.”

## Hyperandrogenism and Metabolic Disorders

### Hyperandrogenism and Insulin Resistance—Mechanisms of Association

**Figure 1.**
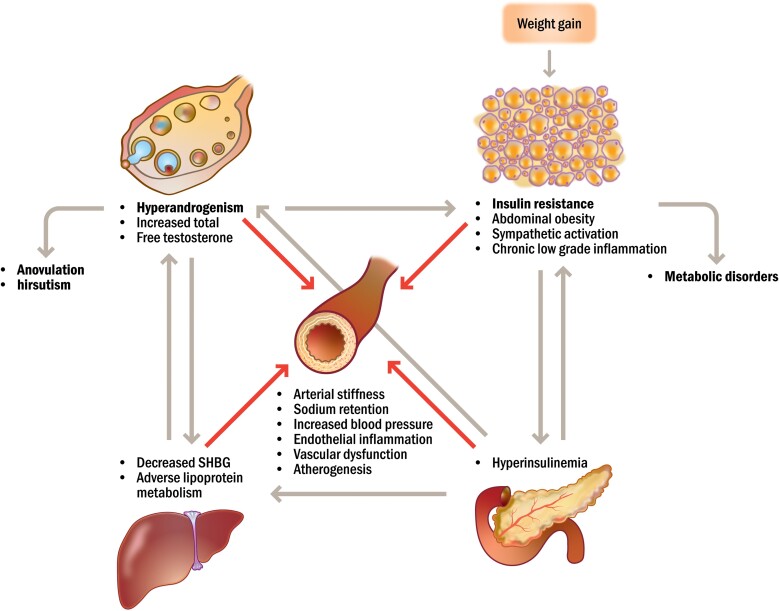
Vicious circle of endocrine and metabolic abnormalities by weight gain leading to aggravating clinical symptoms and increased cardiovascular risk in women with hyperandrogenism. Increased body weight will result in insulin resistance and compensatory hyperinsulinemia. Insulin directly stimulates ovarian biosynthesis of testosterone and inhibits SHBG production in the liver, which will result in higher levels of both total and free testosterone. Hyperandrogenism may in turn induce abdominal fat accumulation and insulin resistance. In this way, the vicious circle could continue and worsen the clinical symptoms of hyperandrogenism and insulin resistance. Furthermore, endocrine and metabolic abnormalities may lead to increased cardiovascular risk. Androgen excess can cause adverse lipoprotein metabolism and atherogenic lipid profile, as well as arterial stiffness and endothelial dysfunction resulting in increased blood pressure. Higher androgens may also induce abdominal adiposity, insulin resistance, and secondary hyperinsulinemia, which can cause sodium retention, increased blood pressure, and chronic low-grade inflammation. In turn, the inflammatory response together with sympathetic activation can lead to endothelial inflammation, vascular dysfunction, and atherogenesis.

Hyperandrogenism and insulin resistance are closely linked and can trigger a vicious circle of aggravated clinical symptoms, including anovulation, hirsutism, and metabolic disorders (41, 42) (Fig. 1). It is still unclear whether elevated testosterone or insulin resistance is the primary abnormality. However, usually weight gain initiates this negative development of endocrine/metabolic abnormalities. An increase in body weight results in insulin resistance and compensatory hyperinsulinemia (17). Insulin may act directly or synergistically with LH to stimulate androgen synthesis from the ovarian theca cells (43), and furthermore, to inhibit SHBG production in the liver (44), resulting in higher levels of both total and free testosterone. Testosterone may in turn promote insulin resistance and accumulation of abdominal fat by different mechanisms, including altered lipolytic activity in visceral and subcutaneous fat tissue (45-49). In this way, the vicious circle may continue and worsen the clinical symptoms of hyperandrogenism and insulin resistance (Fig. 1).

Along with ovarian hyperandrogenism, insulin resistance is a key factor in the pathogenesis of PCOS. The prevalence of insulin resistance in women with PCOS as assessed by the glucose clamp technique is estimated to 65% to 95% (50, 51). Insulin resistance can occur in both normal-weight (52) and overweight individuals with PCOS but is exacerbated by obesity (53). Furthermore, insulin resistance is dependent on PCOS phenotype. The highest occurrence is described in phenotype A with all 3 diagnostic criteria (oligo-anovulation [OA], hyperandrogenism [HA], polycystic ovaries [PCO]) as well as B (OA, HA), followed by C (HA, PCO), and lowest incidence in phenotype D lacking the criterion of hyperandrogenism (OA, PCO) (54-56).

The molecular mechanisms of insulin resistance in PCOS seem to be selective and tissue specific (51). There is evidence of a postreceptor defect in insulin signaling that involves increased serine phosphorylation and decreased tyrosine phosphorylation of the insulin receptor and insulin receptor substrate-1 causing dysfunction of the metabolic pathway but sparing the mitogenic pathway (17, 57). In skeletal muscle, which is the major insulin-dependent peripheral glucose uptake tissue, insulin-resistant mechanisms in PCOS also include mitochondrial dysfunction and intramuscular lipid accumulation (51). In adipose tissue, insulin acts via aldoketo reductase type 3 to stimulate local testosterone production and thereby promotes systemic insulin resistance (58). Furthermore, decreased levels of adiponectin produced by adipose cells result in decreased protein kinase C activity and insulin signaling (59). In ovarian tissue, data suggest selective insulin resistance resulting in impaired insulin-dependent glucose metabolism in granulosa-theca lutein cells but unchanged insulin-stimulated steroid hormone production (60). In addition, there is evidence of defective insulin signaling in the endometrium of women with PCOS as demonstrated by lower endometrial expression of insulin receptor substrate-1 and glucose transporter 4 in comparison to body mass index (BMI)-matched controls (61).

### Obesity

Insulin resistance is associated with accumulation of abdominal fat, which is a common clinical feature even in normal-weight women with PCOS, although not included in the diagnostic criteria (62). In addition to insulin resistance and hyperinsulinemia, other factors may contribute to obesity in PCOS, including testosterone-dependent appetite regulation and lipolytic function, as well as genetic factors, whereas changes in energy expenditure appear less significant (63). According to a systematic review and meta-analysis including 35 studies, the pooled estimated prevalence of obesity in premenopausal women with PCOS is 49%, and the corresponding prevalence of abdominal obesity is 54% (Table 1) (64). A recent systematic review and meta-analysis in peri- and postmenopausal women with PCOS also showed greater BMI, waist circumference, and insulin resistance than controls (Table 2) (9). However, less is known about older women with a history of PCOS. In a small cohort of women with PCOS and controls followed up to a mean age of 81 years, there was no difference in BMI and waist-hip ratio between groups (8). Thus, the enhanced risk of obesity in women with PCOS might diminish at older age.

**Table 1.** Overall estimates of cardiometabolic outcomes in women with PCOS (mainly of reproductive age) compared with controls as based on recent systematic reviews and meta-analyses

Outcome	Estimate
Obesity	RR, 2.77; 95% CI, 1.88-4.10 (64)
Type 2 diabetes	OR, 2.87; 95% CI, 1.37-6.01 (3)
Hypertension	RR, 1.70; 95% CI, 1.43-2.07 (65)
Increased triglycerides	SMD, 0.38; 95% CI, 0.29-0.48 (66)
Increased LDL	SMD, 0.29; 95% CI, 0.20-0.39 (66)
Increased non-HDL	SMD, 0.42; 95% CI, 0.31-0.53 (66)
Metabolic syndrome	OR, 3.35; 95% CI, 2.44-4.59 (67)
Cardiovascular disease	OR, 1.68; 95% CI, 1.26-2.23 (3)
Coronary artery disease	OR, 1.48; 95% CI, 1.07-2.05 (3)
Myocardial infarction	OR, 2.50; 95% CI, 1.43-4.38 (3)
Stroke	OR, 1.71; 95% CI, 1.20-2.44 (3)
Cardiovascular mortality	IRR, 1.26; 95% CI, 1.19-1.34 (3)

References in parentheses.

Abbreviations: HDL, high-density lipoprotein cholesterol; IRR, incident risk ratio; LDL, low-density lipoprotein cholesterol; OR, odds ratio; PCOS, polycystic ovary syndrome; RR, relative risk; SMD, standardized mean difference.

**Table 2.** Evidence of cardiometabolic outcomes in pre- and postmenopausal women with hyperandrogenism (mainly women with PCOS) as based on systematic reviews and meta-analyses

Outcome	Premenopausal nonadjusted for BMI	Premenopausal BMI-adjusted or normal weight groups	Postmenopausal nonadjusted for BMI	Postmenopausal BMI-adjusted
Obesity	+ (64)	--------------	+ (9)	--------------
Type 2 diabetes	+ (3, 68, 69)	+ (3, 52)	+ (9)	= (9)
Hypertension	+ (65, 69)	+ (65, 66)*^a^*	+ (9)	= (9, 65)
Dyslipidemia	+ (66, 69)	+ (66)*^a^*	+ (9)	+ (9)*^b^*
Metabolic syndrome	+ (52, 67, 70, 71)	+ *^a^* (67) = (67)*^c^*	+ (72)	= (72)
Cardiovascular disease	+ (3, 73)	+ (3)	= (9, 73)	= (9)
Coronary artery disease	+ (3)	+ (3)	= (9)	= (9)
Myocardial infarction	+ (3)	+ (3)	+ (9)	= (9)
Stroke	+ (3)	+ (3)	+ (9)	= (9)
Cardiovascular mortality	+ (3, 73)	+ (3)	= (74, 75)*^d^*	= (74, 75)*^d^*

Data are nonadjusted or adjusted for BMI or obtained from normal weight groups only. References in parentheses.

Abbreviations: +, increased; =, neutral; BMI, body mass index; HDL, high-density lipoprotein; PCOS, polycystic ovary syndrome.

^*a*^BMI-adjusted.

^*b*^Only significant decrease in HDL.

^*c*^Normal-weight groups only.

^*d*^Based on 2 separate cohort studies.

Severe hyperandrogenism (ie, virilizing symptoms of endogenous cause, such as ovarian hyperthecosis in a peri- or postmenopausal woman) is clearly associated with insulin resistance and abdominal obesity (20, 76). In fact, insulin resistance and secondary hyperinsulinemia have been suggested to be involved in the pathogenesis of the disorder (10). High levels of insulin can induce stromal luteinization and stimulate androgen production in ovarian theca cells. Usually, the metabolic symptoms of ovarian hyperthecosis are more pronounced than in PCOS and often manifested as acanthosis nigricans, a darkening of the skin in intertriginous areas, which is pathognomonic of insulin resistance (77). Androgen-secreting tumors also seem to be associated with metabolic disorders. Clinical manifestations of insulin resistance and abdominal obesity have been reported for both androgen-secreting ovarian (78) and adrenal tumors (16, 79), respectively. Adrenal tumors may also secrete cortisol in increased amounts, which may contribute to insulin resistance in these patients (1).

Adult patients with CAH have an increased risk of insulin resistance (29%) and obesity (41%) (19, 80, 81). These metabolic complications in CAH may develop at an early age, before puberty (82). In the case of classic CAH, it is not likely that hyperandrogenism as such is the underlying cause of insulin resistance and obesity because androgens are suppressed with glucocorticoid treatment and tend to be low (13). Instead, metabolic complications seem to be related to excess glucocorticoid therapy, as well as familial hereditary factors (82). However, because women with poor disease control may have androgen excess and those with nonclassic CAH are not always treated with glucocorticoids, hyperandrogenism may explain enhanced risk of metabolic complications in some women with CAH (83).

### Type 2 Diabetes

It is well-known that women with PCOS have an increased risk of type 2 diabetes, and PCOS is therefore regarded as a prediabetic condition (3, 68, 69). The most recent systematic review and meta-analysis in the updated “International evidence-based PCOS guideline 2023” based on 41 studies demonstrated an almost 3 times higher risk of type 2 diabetes in women with PCOS with moderate certainty (Table 1) (3). Furthermore, when studies with BMI-matched controls were analyzed separately, women with PCOS still had a higher risk of type 2 diabetes than controls (odds ratio [OR], 3.04; 95% CI, 2.06-4.49) (Table 2) (3). Similarly, another systematic review and meta-analysis in exclusively nonobese women with PCOS (22 studies) reported increased prevalence of type 2 diabetes compared with nonobese healthy controls (52). The risk by ethnicity has shown the highest prevalence for Asia and lowest for Europe (68). Previous studies on US populations demonstrated that up to 40% of women with PCOS have impaired glucose tolerance and 10% develop type 2 diabetes by their fourth decade (84). In European populations, the prevalence rates appear to be lower (68), and a large Nordic cohort showed no enhanced prevalence of type 2 diabetes in normal-weight women with PCOS (85). A systematic review and meta-analysis of peri- and postmenopausal women with PCOS (13 studies) showed an increased OR for diabetes; however, when adjusted for BMI, there was no longer any increased rate (Table 2) (9). Taken together, PCOS in women of reproductive age is an independent risk factor for type 2 diabetes, whereas the risk for diabetes in postmenopausal women with hyperandrogenism is dependent on increased BMI.

Ovarian hyperthecosis is characterized by virilizing symptoms but also, as already mentioned, manifestations of insulin resistance (abdominal obesity, acanthosis nigricans) and, consequently, there is an enhanced occurrence of type 2 diabetes (20, 86). Because there are only small case series in the literature, there is no estimated prevalence of diabetes, but most patients have been reported to have this diagnosis (20, 78, 86-88). Type 2 diabetes also seems to be a common comorbidity in women with androgen-secreting tumors. Six of 13 women with androgen-secreting ovarian tumors were reported to have type 2 diabetes (78). However, the corresponding occurrence of type 2 diabetes in adrenal tumors is not known.

A Swedish and a Korean nationwide register-based study have both shown that CAH in women is associated with an increased risk of type 2 diabetes compared with age- and sex-matched controls (OR, 2.8-3.0) (80, 89). However, the studies were not adjusted for BMI. The underlying cause is not established but is generally thought to be related to overtreatment with glucocorticoids to suppress androgens (82).

## Hyperandrogenism and Cardiovascular Risk Factors

### Hyperandrogenism and Cardiovascular Risk Factors—Mechanisms of Association

Although it has long been known that hyperandrogenic conditions are related to increased occurrence of cardiovascular risk factors, including hypertension, hyperlipidemia, and the complete metabolic syndrome (17, 18, 21), the underlying mechanisms are not completely understood. Figure 1 summarizes potential mechanisms underlying enhanced cardiovascular risk in women with hyperandrogenism. One factor is androgen excess, which independently promotes the development of atherogenic lipid profile by a direct effect on the lipoprotein metabolism in the liver (90), as well as hypertension related to effects of androgens on the vascular system including arterial stiffness and endothelial dysfunction (91-94). Furthermore, higher testosterone induces abdominal adiposity, insulin resistance, and secondary hyperinsulinemia (62), which in turn have been associated with sodium retention and increased blood pressure (95), impaired vascular function (96), and chronic low-grade inflammation (97). The inflammatory response of various cytokines such as high-sensitivity C-reactive protein, as well as adipokines including adiponectin (98-100), induce formation of reactive oxygen species as markers of oxidative stress and metabolic dysfunction (101, 102). This immune response, strongly related to abdominal obesity, has been implicated in atherogenesis by causing endothelial inflammation and vascular dysfunction (99, 100, 102). Moreover, enhanced sympathetic nervous system activity leading to increased blood pressure and systemic inflammation has also been suggested to be involved in the etiology of cardiovascular risk in women with hyperandrogenism (103).

### Hypertension

Data support an association between PCOS and hypertension (9, 65, 66, 69). A recent systematic review and meta-analysis (38 studies) demonstrated that PCOS in women of reproductive age is associated with higher blood pressure, regardless of BMI, than controls (Table 2) (66). Furthermore, a previous systematic review and meta-analysis including 30 studies reported a significantly higher pooled relative risk of hypertension in reproductive-aged women, but not in postmenopausal women, with PCOS after BMI adjustment (Table 1) (65). In agreement, a meta-analysis of women with PCOS of peri- and postmenopausal age (14 studies) showed an overall increased OR of hypertension but when restricting the analysis to studies with BMI-matched controls, there was no longer an enhanced risk of hypertension in women with PCOS (Table 2) (9). It appears that PCOS is an independent risk factor for hypertension in women of reproductive age, but in older women obesity plays a critical role for this association.

Almost all cases of ovarian hyperthecosis are reported to have hypertension (20, 76, 87, 88). After bilateral oophorectomy, which is the recommended treatment for a postmenopausal woman, metabolic symptoms including hypertension may improve but do not normalize completely, indicating that metabolic disturbances probably are intrinsic and not dependent on hyperandrogenism (1, 10). Androgen-secreting tumors are also associated with hypertension, but as with ovarian hyperthecosis, scientific support is based on case reports (16, 104, 105). In case reports of androgen-secreting tumors, improvements in blood pressure and reduced doses of antihypertensive drugs have been reported after surgical treatment (105).

In addition to increased risk of type 2 diabetes, women with CAH also have a higher risk of hypertension as shown in population-based register studies (OR, 2.9-4.1), as well as longitudinal cohort studies (19, 80, 82, 89). Furthermore, a systematic review and meta-analysis demonstrated higher systolic and diastolic blood pressure in patients with CAH than controls (81). However, the quality of evidence was assessed as low, and data were not available to perform separate analyses with respect to sex, BMI, and medication (81). Excessive treatment with glucocorticoids and mineralocorticoids, as well as uncontrolled androgen excess have been suggested as contributing factors. A longitudinal study identified suppressed androstenedione levels, reflecting excess glucocorticoid therapy, as a risk factor for hypertension in adult patients with CAH (82).

### Dyslipidemia

Women with PCOS have a higher risk of adverse lipid profile (52, 66, 69). A recent systematic review and meta-analysis (38 studies) comparing BMI-matched groups of women with PCOS and controls for different BMI categories demonstrated that triglycerides, low-density lipoprotein cholesterol and non–high-density lipoprotein (HDL) cholesterol were clearly increased in women with PCOS of reproductive age, independent of BMI category (Table 1) (66). However, a Nordic cohort study showed that lipid profiling in women younger than age 35 years with PCOS and assessed as having low cardiovascular risk rarely changed the clinical care according to guidelines (106). In contrast, women with PCOS who also have type 2 diabetes and/or hypertension and are categorized as having high cardiovascular risk should be monitored with lipid profiling for potential treatment (106). Peri- and postmenopausal women with PCOS also have enhanced risk of adverse lipid profile, specifically decreased HDL and higher triglycerides, as demonstrated in a systematic review and meta-analysis (9). However, subgroup analysis of BMI-matched groups (13 studies) showed that only reduction in HDL in women with PCOS remained significant (Table 2) (9). Overall, PCOS is associated with dyslipidemia, which is independent on BMI for women of reproductive age. However, in peri- and postmenopausal women, adverse lipid profile is largely dependent on BMI except for low HDL.

Dyslipidemia is a common condition also in women with ovarian hyperthecosis (1, 20, 87, 88). Barth et al described 4 cases in which all had dyslipidemia (20). They were treated with GnRH agonist, which was highly effective in reducing the high testosterone levels and hirsutism, but 6 months of treatment had no effect on the lipid profile (20). Likewise, an adverse lipid profile has been reported in cases of androgen-secreting tumors (105).

CAH is associated with an elevated risk of dyslipidemia (19, 80, 82, 89). Population-based register studies from Sweden and Korea reported increased OR of about 3.0 for dyslipidemia in women with CAH (80, 89). Furthermore, an earlier British longitudinal cohort study showed that 48% of women with classic CAH had hypercholesterolemia (19). However, the influence of obesity, CAH phenotype, and genotype, as well as treatment with glucocorticoids and/or mineralocorticoids on the lipid profile has not been studied (19, 80, 82, 89).

### Metabolic Syndrome

PCOS has been associated with metabolic syndrome and all its components (ie, abdominal obesity, dyslipidemia, hyperglycemia, and hypertension) (52, 67, 70, 71). A systematic review and meta-analysis (27 studies) showed that women of reproductive age with PCOS have an overall 3 times higher risk of metabolic syndrome (Table 1) (67). This enhanced risk remained in BMI-matched analyses of overweight or obese women, whereas lean women had no increased risk (Table 2) (67). It was shown that metabolic features (eg, waist circumference, lipids) contributed to the risk but not indices of hyperandrogenism (testosterone, SHBG) (67). On the other hand, higher prevalence of metabolic syndrome has been associated with hyperandrogenic phenotypes of PCOS (A, B, and C) than the normoandrogenic phenotype (D) (107). The incidence of metabolic syndrome was investigated across menopausal transition in the longitudinal Women's Health Across the Nation study (72). Unadjusted results showed higher rates of metabolic syndrome in women with a history of androgen excess and menstrual irregularity; however, after adjustment for covariates including BMI, there were no longer any increased incidence rate in these women compared with normoandrogenic women (72). Smoking and obesity were the strongest predictors of metabolic syndrome (72). It appears that postmenopausal women with PCOS are at lower metabolic risk than premenopausal women with PCOS, whereas metabolic syndrome in women of reproductive age is associated with obesity.

The occurrence of metabolic syndrome in patients with ovarian hyperthecosis is not known, although the majority of reported cases have several components of the metabolic syndrome, including diabetes mellitus, hypertension, and hyperlipidemia (1, 20, 76, 87, 88). Metabolic syndrome is also not known for androgen-secreting tumors, but the clinical similarity between virilizing ovarian tumors and ovarian hyperthecosis is substantial, including the presence of obesity and type 2 diabetes (78, 105). Furthermore, enhanced cortisol secretion in association with androgen-secreting tumors of adrenal origin may mimic Cushing syndrome and its metabolic manifestations (1, 16).

Women with CAH have a higher prevalence of the components of metabolic syndrome that that include obesity, type 2 diabetes, hypertension, and hyperlipidemia (19, 80-82, 89). One study also investigated the complete metabolic syndrome in patients with CAH and reported a prevalence of 40% (82). Obesity, as well as glucocorticoid and mineralocorticoid overtreatment were related to metabolic risks (82).

## Hyperandrogenism and Cardiovascular Disease and Mortality

### Hyperandrogenism and Cardiovascular Disease—Mechanisms of Association

The overall association between clinical hyperandrogenism and higher prevalence of cardiovascular risk factors is well established (Table 2). Hyperandrogenism in women of reproductive age is also associated with subclinical markers of atherosclerotic cardiovascular disease, such as arterial stiffness (91), carotid intima media thickness (108), coronary artery calcification (109), and endothelial dysfunction (110). How is this translated to the risk of cardiovascular disease and mortality in these women?

**Figure 2.**
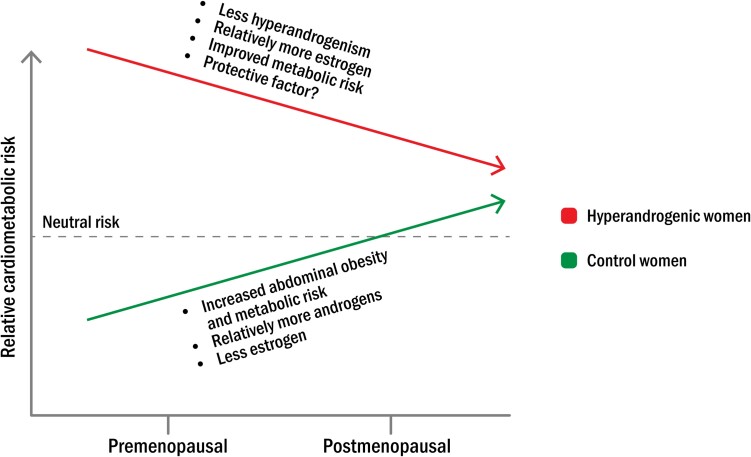
Hypothesis of changed cardiometabolic risk in women with hyperandrogenism from premenopausal age to postmenopausal age. Normal menopausal transition is associated with increased abdominal obesity and cardiometabolic risk, which could be related to a physiological shift in hormonal balance from estrogen dominance to a relative predominance of androgens. In contrast, women with hyperandrogenism experience fewer androgenic symptoms and have lower metabolic risk after menopause in association with a decline in androgens and relatively more estrogen.

In general, cardiovascular disease usually develops in women after menopause independently of age, since estrogen is considered to have protective effects on the cardiovascular system (27, 28). Women of postmenopausal status also have a higher risk of abdominal obesity, insulin resistance, and type 2 diabetes compared with premenopausal women (111). Diabetes in turn causes almost a 5-fold higher risk of myocardial infarction in women (112). Normal menopausal transition is related to a sex hormone change leading to relative androgen dominance, which may play a role in increased cardiometabolic risk. In contrast, women with hyperandrogenism such as PCOS rather become less androgenic after menopause (23, 113, 114). Furthermore, there is no support for further weight gain and metabolic deterioration in women with PCOS because risk factors for diabetes may even improve after menopause (115-118). Figure 2 illustrates a hypothesis of changed cardiometabolic risk in women with hyperandrogenism after menopause in comparison with nonhyperandrogenic women.

There is still a debate whether hyperandrogenism in women leads to an enhanced risk of cardiovascular events and mortality. The state of evidence today is summarized here.

### Cardiovascular Disease

Composite cardiovascular disease (angina, myocardial infarction, coronary artery angioplasty, revascularization, peripheral vascular disease, stroke, transient ischemic accident, cardiovascular-related deaths) was investigated in a meta-analysis in the recently updated International PCOS guideline 2023 (3). The meta-analysis, based on 10 cohort studies, reported a significantly increased OR of composite cardiovascular disease in women with PCOS with low or very low certainty because all the evidence was generated from observational studies and of relatively short-term follow-up (Table 1) (3). The majority of the studies (9 of 10) were BMI-matched or adjusted for BMI and primarily based on women of reproductive age with less-defined PCOS status. In contrast, women of exclusively nonreproductive aged ≥45 years with PCOS have similar OR of cardiovascular disease as controls in recent systematic reviews and meta-analyses (Table 2) (9, 73). Taken together, the data suggest that the increased risk of cardiovascular events in women with PCOS might decline over time to normalize at postmenopausal age. Further studies are needed to examine the validity of such hypothesis.

For ovarian hyperthecosis and androgen-secreting tumors, there are no published reports on the overall risk of cardiovascular disease or other cardiovascular outcomes, as discussed later, because these hyperandrogenic conditions are rare. Nevertheless, case reports clearly show associations with cardiometabolic risk factors (1, 10, 16, 20, 76, 78, 105). Importantly, metabolic symptoms persist to a large extent after GnRH treatment in ovarian hyperthecosis (1, 10, 20), as well as after surgical normalization of hyperandrogenism in women with ovarian androgen-secreting tumors (119). The consequence of persistent metabolic disturbances for the long-term risk of cardiovascular disease in these women is unclear.

CAH is associated with increased cardiovascular morbidity (80, 89). A Swedish population-based cohort study of 335 women reported a nearly 4 times higher risk of cardiovascular disease (OR, 3.9) compared with matched controls (80). A Korean epidemiological study also showed a higher risk of cardiovascular disease in women aged ≥40 years than controls (OR, 2.3) (89). Obesity was more prevalent in patients with CAH and cardiovascular disease than those without but could not fully explain the enhanced cardiovascular morbidity in the Swedish women with CAH (80, 82).

### Coronary Artery Disease and Myocardial Infarction

The latest International PCOS guideline 2023 further performed meta-analyses on composite coronary artery disease (angina, myocardial infarction, coronary artery angioplasty, revascularization) based on 7 cohort studies and on myocardial infarction including 9 cohort studies (3). Women with PCOS have increased OR for both composite coronary artery disease and myocardial infarction with low to very low certainty of evidence (Table 1). Most of the studies were on reproductive aged women with PCOS and controls, matched or adjusted for BMI. A meta-analysis on women with PCOS of peri- or postmenopausal age, showed similar OR for coronary artery disease as controls but increased OR (2.51) for myocardial infarction (3 studies) (Table 2) (9). However, when restricting meta-analysis to studies with BMI-matched controls, there was no longer any enhanced risk of myocardial infarction in peri- or postmenopausal women with PCOS (Table 2) (9). Overall, meta-analyses support that women of reproductive age with PCOS have higher risk of coronary artery disease and myocardial infarction. However, in women of nonreproductive age with PCOS, there is no evidence of increased risk of composite coronary artery disease, and the enhanced risk of myocardial infarction in these women is explained by weight excess.

In patients with CAH, a systematic review and meta-analysis showed higher occurrence of carotid intima thickness (81). However, a Swedish population-based cohort study reported similar risk as controls for acute coronary syndrome (heart attack and unstable angina) in women with CAH (80). No other published study on this specific outcome in CAH was found in the literature.

### Stroke

Women with PCOS have increased OR of stroke (ischemic and hemorrhagic cerebrovascular accidents) according to a meta-analysis in the updated International PCOS guideline 2023 (Table 1) (3). The meta-analysis was based on 485 365 women in 10 cohort studies, of which 5 were longitudinal, and 8 of 10 studies were matched or adjusted for BMI and women were primarily of reproductive age. The overall certainty of evidence was judged as low to very low. Increased OR of stroke (OR, 1.75) was also reported in a meta-analysis of exclusively peri- and postmenopausal women with PCOS (9). The meta-analysis based on 4 studies was not adjusted for BMI; however, when comparing women with PCOS and controls with similar BMI, the significant difference disappeared (Table 2) (9). Thus, the higher risk of stroke in peri- and postmenopausal women with PCOS seems to be dependent on obesity.

In women with CAH (n = 335), OR of stroke was similar to controls in a Swedish population-based cohort study (80). However, in a Korean epidemiological study, OR for stroke was significantly increased (OR, 2.3) in women ≥40 years (n = 475) (89). Data did not enable analysis of obesity and/or glucocorticoid over- or undertreatment as underlying cause.

### Cardiovascular Mortality

The incident risk ratio of cardiovascular mortality was reported to be significantly increased in women with PCOS in a meta-analysis of low certainty of evidence in the International PCOS guideline 2023 (Table 1) (3). The meta-analysis was based on 4 cohort studies judged as moderate risk of bias, the studies were all BMI-matched or BMI-adjusted, half of them were on women of premenopausal age and the other 2 on postmenopausal or older women (3). The 2 separate small cohort studies on postmenopausal women with PCOS showed no difference in cardiovascular mortality compared with BMI-matched controls (Table 2) (74, 75).

Mortality in CAH has been investigated in 2 population-based studies. A Swedish study showed an overall enhanced mortality rate in women with CAH compared with controls (hazard ratio, 3.5) (120). Most cases were related to adrenal crises but 32% had a cardiovascular cause. A Korean study also reported an increased all-cause mortality rate (hazard ratio, 1.6) in patients with CAH; however, the underlying causes were not investigated (89). In addition, a study based on UK primary care data showed a 6-fold higher risk of all-cause mortality in women with CAH compared with a reference group, but no further analysis of specific causes was performed (121).

## Summary and Conclusion

The association between clinical hyperandrogenism and cardiometabolic risk factors, such as type 2 diabetes, hypertension, dyslipidemia, and metabolic syndrome in women of reproductive age is well established. Furthermore, recent scientific evidence also supports higher risk of cardiovascular disease that includes coronary artery disease, myocardial infarction, and stroke, as well as cardiovascular mortality in women with hyperandrogenism of reproductive age. These associations are independent of obesity because data based on BMI adjustments or comparisons between BMI-matched groups still show an increased risk in women with hyperandrogenism compared with controls. In postmenopausal women with clinical hyperandrogenism, there is also evidence of higher prevalence of cardiometabolic risk factors that include type 2 diabetes, hypertension, dyslipidemia, and metabolic syndrome. However, in contrast to premenopausal women, the associations in postmenopausal women are highly dependent on obesity. Only reduced HDL remains significant after BMI adjustment. Furthermore, prevalence of cardiovascular disease as a whole and coronary artery disease are similar in postmenopausal women with hyperandrogenism as controls. Although myocardial infarction and stroke as specific outcomes are increased in postmenopausal women with hyperandrogenism, these associations disappear after BMI adjustment. Taken together, there is scientific support for higher prevalence of both cardiometabolic risk factors as well as cardiovascular disease independent of BMI in premenopausal women with clinical hyperandrogenism. These risks decline with aging, and remaining risks in hyperandrogenic women of postmenopausal age are mainly explained by obesity. Although these conclusions are based on systematic reviews and meta-analyses, it must be taken into account that data are of low certainty and on women with PCOS only.

How do we explain reduced cardiometabolic risks in postmenopausal women with clinical hyperandrogenism? It can only be speculated that hyperandrogenic and nonhyperandrogenic women approach each other regarding endocrine balance and metabolic constitution after menopause when women with hyperandrogenism become less hyperandrogenic at the same time as nonhyperandrogenic women tend to develop androgen dominance related to abdominal obesity during normal menopausal transition (Fig. 2). Still, obesity is an important factor for increased cardiovascular risk in postmenopausal women with hyperandrogenism compared with controls. Although consideration should be given to the fact that many affected individuals perceive obesity as stigmatizing, the health care system must not disregard the importance of obesity as a health risk, not at least in postmenopausal women.

In recent years, we have gained increased knowledge about cardiometabolic risks in women with clinical hyperandrogenism. This knowledge is of utmost importance considering that cardiovascular disease is the most common cause of death in women globally, and measures should focus on the prevention of risk factors. Still, more data are needed on, particularly, cardiovascular disease and mortality from larger long-term follow-up studies in women with PCOS of specified phenotypes. In other patient groups, more research is needed about the role of insulin resistance for development of severe hyperandrogenism as in ovarian hyperthecosis, as well as the impact of over- or undertreatment with glucocorticoids and mineralocorticoids for cardiometabolic risks in women with CAH.

## Data Availability

Data sharing is not applicable to this article as no datasets were generated or analyzed during the current study.

## Abbreviations

BMI: body mass index
CAH: congenital adrenal hyperplasia
FAI: free androgen index
HA: hyperandrogenism
HDL: high-density lipoprotein
OA: oligo-anovulation
OR: odds ratio
PCO: polycystic ovary
PCOS: polycystic ovary syndrome
